# A Strategy toward Realizing Ultrashort Channels and Microstructures Array by Piezoelectric Inkjet Printing

**DOI:** 10.3390/nano9111515

**Published:** 2019-10-24

**Authors:** Jianqiu Chen, Liao Gan, Zhipeng Pan, Honglong Ning, Zhiqiang Fang, Hongfu Liang, Ruiqiang Tao, Wei Cai, Rihui Yao, Junbiao Peng

**Affiliations:** 1 State Key Laboratory of Luminescent Materials and Devices, Institute of Polymer Optoelectronic Materials and Devices, South China University of Technology, Guangzhou 510640, China; c.jianqiu@mail.scut.edu.cn (J.C.); 201530291429@mail.scut.edu.cn (H.L.); c.w01@mail.scut.edu.cn (W.C.); psjbpeng@scut.edu.cn (J.P.); 2 Air Force Representative Office in Zunyi District, Zunyi City 563000, China; 15985282924@139.com; 3 Guizhou Meiling Power Supply Co., Ltd., Zunyi City 563000, China; pzpmlhg2004@126.com; 4 State Key Laboratory of Pulp and Paper Engineering, South China University of Technology, Guangzhou 510640, China; fangzq1230@126.com; 5 South China Academy of Advanced Optoelectronics, South China Normal University, Guangzhou 510006, China; taoruiqiang@m.scnu.edu.cn; 6 Guangdong Province Key Lab of Display Material and Technology, Sun Yat-sen University, Guangzhou 510275, China

**Keywords:** inkjet printing, inkjet etching, “coffee-ring” effect, homogeneous, microstructures

## Abstract

Inkjet printing has been proved to be a powerful tool in the cost-effective ambient deposition of functional materials for the fabrication of electronic devices in the past decades. However, restricted by equipment and inks, the feature size of printed dots or lines with conventional inkjet printing is usually limited to several tens of micrometers, which could not fit the requirements for the fabrication of large-area, high-resolution microscale, even nanoscale, structures. Therefore, various technical means were developed for breaking the equipment limits. Here, we report a strategy for realizing ultrashort channels and homogeneous microstructures arrays by a conventional piezoelectric inkjet printing technique without any additional pre-mask process on the substrate. This strategy extends application of piezoelectric inkjet printing technique to biological and technological areas.

## 1. Introduction

Inkjet printing has been utilized in developing thin-film electronic devices in the past decades on account of its merits of direct patterning, cost-effective, environment-friendly and mass production [1,2,3,4]. With suitable inks and appropriate processes, this technology could enable high-performance field-effect transistors [5], inverters [6], organic light-emitting diodes (OLEDs) [7], solar cells [8,9], photodetectors [10], or rollable electronics. However, a well-known phenomenon called the “coffee-ring” effect, which is induced by the radial capillary flow, usually blocks the way to obtain uniform films [11]. However, on the other hand, the coffee-ring effect offers us a novel method for realizing specific structures such as short channels or banks [12,13,14]. Layani et al. have obtained transparent conductive patterns utilizing the coffee-ring effect by adjusting various inks and printing parameters [15]. Li et al. have realized inkjet-printed metal-oxide thin-film transistors with short channels using coffee-ring-defined banks as the barriers [16]. Chen et al. have fabricated microlens arrays by creating microwell structures based on the “coffee ring” effect [17]. Li et al. have assembled large-scale transparent circuit arrays using the “coffee ring” effect by simply pipetting gold nanoparticles suspension onto a polydimethylsiloxane (PDMS) nanofilm-patterned substrate with distinct hydrophilic/hydrophobic interactions [18]. In short, emerging as an unconventional means, the “coffee ring” effect, which could usually bring unexpected results, inspires researchers to develop new approaches in nano/microscale application [19,20,21,22]. 

Inkjet printing is usually used for depositing a functional layer in a precise location. More than that, a special technique called “inkjet etching” has broadened its applications [23,24,25]. The mechanism of “inkjet etching” is as follows. A droplet which is ejected from an inkjet printer dissolves the substrate locally [22,26]. As a result, with the impact of coffee-ring effect, a hole is formed by redeposition of the dissolved substrate at the contact line. In addition, if the printed ink is not just a pure solvent but with solutes, it can generate a ring-like hole with a uniformly deposited solute at the periphery of the droplet. As we know, the resolution of direct inkjet printing technology is generally determined by the drop size and the interaction between the droplet and substrate [27]. Without any treatment or pre-graphical processing, the resolution limitation of direct piezoelectric inkjet printing technology is about 30–50 μm [28]. For breaking the limitation, continuous efforts have been made to reduce the channel length and linewidth by inkjet printing. For example, Zhang et al. have successfully fabricated a series of conductive patterns with 5–10 μm line widths on hydrophilic glass substrates. Wang et al. have developed a method named “coffee ring lithography” to successfully fabricate channels of inkjet-printed polymer microarrays or libraries, which have a length with a high resolution of 1–2 µm [29]. In particular, the combination of “inkjet etching” and “coffee-ring effect” could often bring precise structures and high-quality microstructure patterns than direct inkjet printing.

In this paper, we report on the investigation of a kind of silver ink (a mixture of silver nanoparticles, a solvent, a stabilizer, and a dispersant) deposited on an uncross-linked poly (vinyl phenol) (PVP) substrate. The resulting structures are investigated as a function of the deposited drop space. Moreover, the formation process of the microstructures is discussed in detail. In fact, the combination of solvents, solutes, and a substrate for etching may bring us a new strategy for materials deposition. In addition to that, it enables the inkjet printing technique to be used in large-area, high-resolution applications using this strategy and always bring us unexpected symmetry and uniformity. This discussion includes a detailed microcosmic investigation of the structure of the holes using a confocal laser scanning microscope.

## 2. Experimental

After the glass substrate was washed by isopropyl alcohol, deionized water, and isopropyl alcohol with ultrasonic cleaning sequentially, a PVP layer was coated on the pre-cleaned glass from a solution of PVP (Sigma-Aldrich, St. Louis, MO, USA) and poly (melamine co-formaldehyde) (PMF, St. Louis, MO, USA) (20:1 wt%) in ethylene glycol (EG, Sigma-Aldrich, St. Louis, MO, USA) and then cured by ultraviolet light for 3 min to obtain a uniform uncross-linked PVP layer. After that, the silver ink (DGP 40TE-20C, Advanced Nano Products, Bugang-myeon, Sejong-si, Korea) with a solvent of triethylene glycol monoethyl ether (TGME) was deposited on the PVP layer by a piezoelectric inkjet printer (Fujifilm Dimatix, DMP2800, Santa Clara, CA, USA) using a 10pl cartridge with a 21 μm nozzle. Finally, the samples were cured on a hot plat at 200 °C for 10 min.

A step profiler (Dektak XT, Bruker, CA, USA) was utilized for cross-sectional scanning across the prepared samples. The optical microscopy images of printed electrodes were obtained by a Nikon Eclipse E600 POL with a DXM1200F digital camera (DXM1200F, Nikon, DeWitt, IA, USA). A confocal laser scanning microscope (OLYMPUS OLS5000, OLYMPUS, Tokyo, Japan) was used to determine the microscopic surface topography.

## 3. Results and Discussions

**Figure 1:**
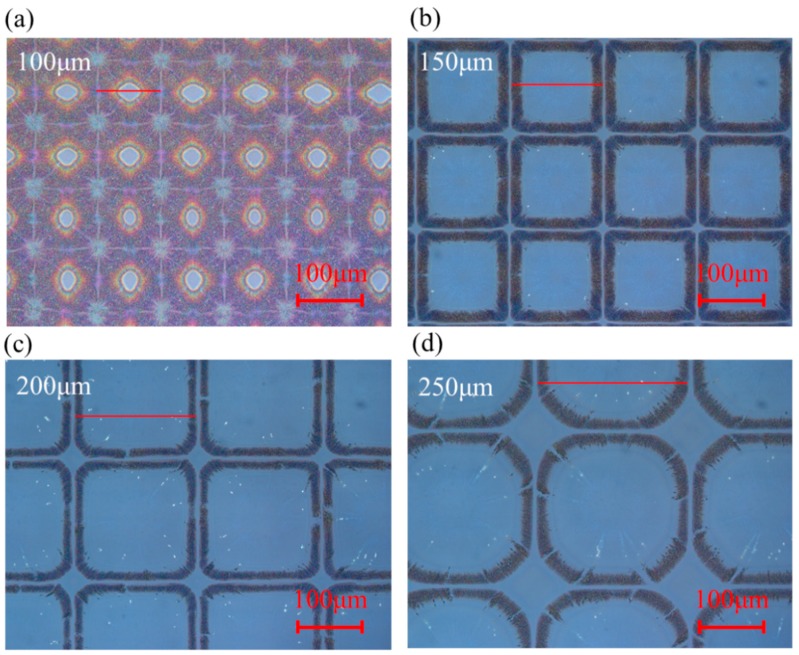
Optical microscopy images of obtained microstructures with different drop spaces: (**a**) 100 μm, (**b**) 150 μm, (**c**) 200 μm, and (**d**) 250 μm.

Figure 1a–d shows the inkjet-printed droplets formed ring-liked microstructures on the pre-coated uncross-linked PVP substrate, and the droplet spaces are 100, 150, 200, and 250 μm, respectively. With the increasing of the droplet spaces, the ring-liked structures also grew. The size of the microstructures is approximately equal to the droplet space, and no apparent intersection was found between adjacent rings. Moreover, ultrashort channels were formed between each ring. In addition, the corners of the microstructures became smoother with the increasing of the droplet spaces. It should be mentioned that a series of cracks emerged on the continuous ring structure due to the consumption of the solvent in the outward diffusion process.

**Figure 2:**
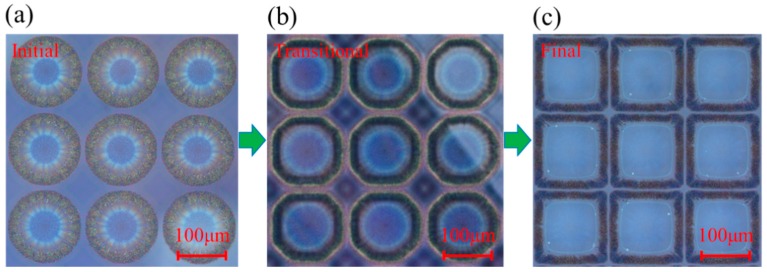
The dynamic formation process of obtained microstructure (left to right): (**a**) the initial state, (**b**) the transitional state, and (**c**) the final state.

The size of the ring structures was expected to depend on a combination of four factors: the droplet space, the spreading of the solvent on the pre-coating substrate, the evaporation rate of the solvent, and the dissolution rate of the polymer. It is worth noting that the dissolving process is a dynamic process; here, we captured the transformation process of the dynamic process. Figure 2 reveals the dynamic process of fabricated rings array in PVP using silver nanoparticle ink to locally dissolve the polymer material, and the drop space was set to 150 µm. As we can see, Figure 2a presents the initial state of the droplet, the droplet takes the form of a regular circle, and each drop is relatively independent. The ring-like structure formed immediately as soon as the droplet was deposited on the substrate. Because of the radial capillary flow induced by the heterogeneous evaporation, the solute migrated to the edge of the droplet rapidly and resulted in a big hole in the center of the droplet. As time went on, the solvent in the droplet dissolved the substrate and the ring expanded along a radial direction, as shown in Figure 2b. It is worth noting that the holes had a more octagon shape rather than a circle shape. The boundary stopped sliding toward to the radial direction, when the circles were close to each other, but along with the direction of the tangent direction. Finally, regular, homogeneous square microstructures arrays were formed on the substrate with ultrashort channels between each unit, as a typical example shown in Figure 2c.

**Figure 3:**
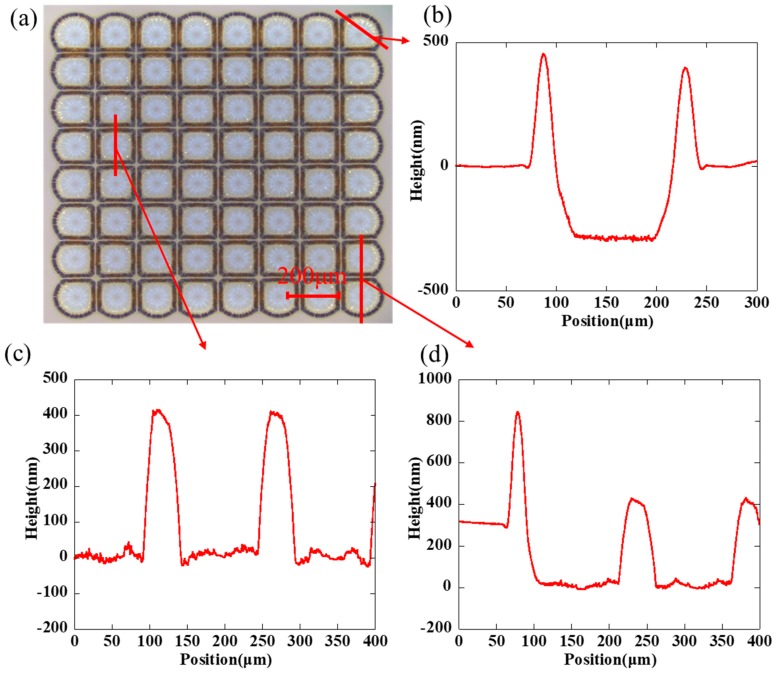
(**a**) Orderly array by inkjet etching and the cross-sectional profile scanning at different areas by a step profiler: (**b**) along the radial direction, (**c**) the inner adjacent rings, (**d**) the adjacent rings at the edge. The droplet space is 150 μm.

**Figure 4:**
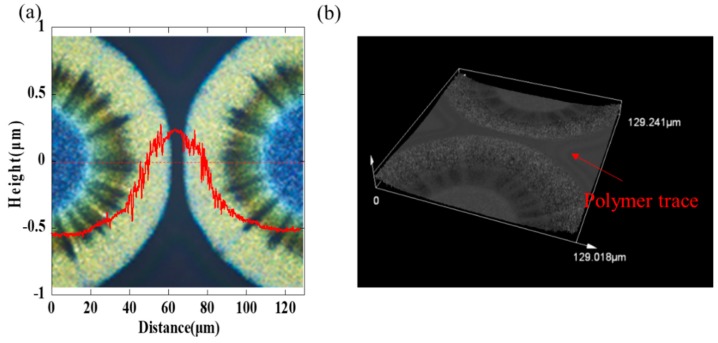
(**a**) The gap between adjacent droplets and the profile of the red line in the center; (**b**) obvious polymer trace around the ring structure. Images were obtained by the confocal laser scanning microscopy.

Figure 3a shows an orderly array by inkjet etching and the cross-sectional scanning by a step profiler, and the droplet space is 150 μm. As we can see clearly in Figure 3b–d, the height of the polymer layer is about 300 nm and the height of silver ring is about 400–600 nm. The size of the hole is about 100 μm × 100 μm and the width of the ring is about 20 μm. The peripheral structure of the array was irregular because no barrier limited its outward expansion, so it was deposited as an arc-shaped structure at the periphery and a square shape inside. The bottom of the hole was relatively flat within the resolution of the step profiler, indicating that the solvent in the droplet completely penetrated the polymer layer. However, there still existed few visible residues in the holes from the optical microscopy image, which may be ascribed to residual silver nanoparticles adsorbed by the surface energy. The cross-section indicates regular sharp peaks which separated holes. Furthermore, it is noted that the adjacent holes were not mixed together. The ultrashort channel formed among these microstructures. We can see clearly in Figure 4a that the gap was not a concave shape, but a convex structure. In addition, in Figure 4b, an obvious polymer trace around the deposited ring was founded, which proved that the gap would be filled with the pre-coated polymer. 

**Figure 5:**
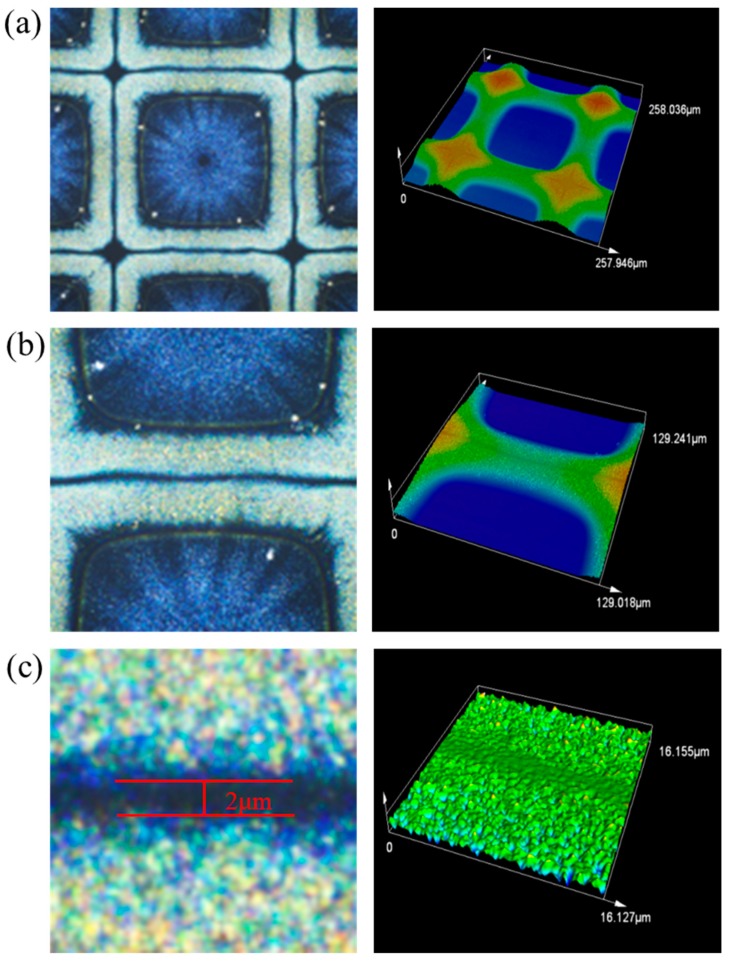
Confocal laser scanning microscopy images and 3D pictures captured under different magnifications: (**a**) 1127×, (**b**) 2254×, and (**c**) 18,031×.

**Figure 6:**
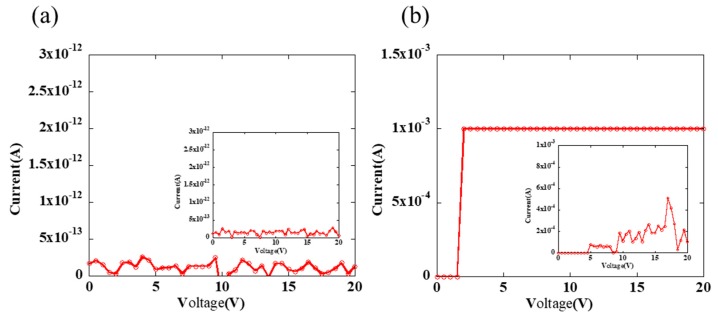
The current as a function of voltage when the probes stabbed at the opposite angles of a single ring: (**a**) annealing at 200 °C for 30 min, and (**b**) annealing at 400 °C for 30 min. The drop space is 150 μm. Insets are the shorting tests results between two silver traces.

The detailed images captured by the confocal laser scanning microscopy are shown in Figure 5, and the magnifications of all the pictures are 1127×, 2254×, and 18,031×, respectively. The corner among the adjacent 4 square structures was not empty, which is the same as the gap, but it was filled with polymer and exhibited a convex structure. There was no contact between adjacent structures in the image even if the channel was not straight. Additionally, the final gap is about 2 μm, which could not be stably directly printed by the piezoelectric inkjet printing technology without extra processes. In addition, we have measured the conductivity of the obtained single ring and adjacent rings. As shown in Figure 6a, the silver rings show non-conductive properties when they were annealed at 200 °C for 30 min. We presumed that the silver nanoparticles were surrounded by polymers because PVP is a good capping agent, and the annealing process promoted the polymer to form the cross-linked structure. The inset in Figure 6a shows no shorting between two adjacent rings. However, when they were annealed at 400 °C for 30 min, the silver rings show good conductivity due to the decomposition of polymer, as shown in Figure 6b. The current between two adjacent rings increased when the rings were annealed at 400 °C for 30 min, which may be ascribed to the leakage current induced by a high voltage, and there was no obvious shorting, as shown in the inset in Figure 6b.

**Figure 7:**
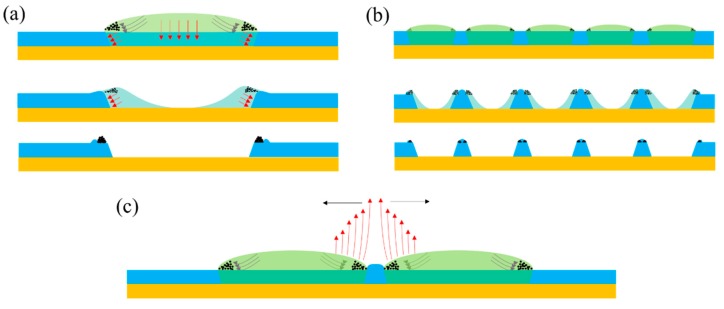
The dynamic formation process of obtained microstructure: (**a**) a single dot, (**b**) a dots array, and (**c**) a repulsive force model to prevent adjacent droplets from coalescing.

Because the shape of the etched structures always reflects the state of the solute and solvent in the droplet during the etching process, these microstructures are certainly the result of the interaction of adjacent droplets and the substrate. The strong evaporation due to the high surface-to-volume ratio at the edge of the droplet induced the solvent compensation from center to the edge. The dissolution of the PVP promoted the slide of the triple-phase contact line (TCL); the TCL stopped sliding toward to the radial direction without coalescence, but along the direction of the tangent direction. Pasquale et al. thought the repulsion may originate from the rapid evaporation of solvent and the resulting flux of solvent vapor, or from the thermal-gradient-driven Marangoni convection inside the droplets, causing a repulsive air flow between them [19]. Here, we built a simple model to clarify the formation process. Figure 7a,b exhibit the cross-sectional diagrams of the single and multiple droplets deposited on the pre-coated PVP substrate, finally resulting in inerratic microstructures. As soon as the droplet contacted the substrate, the solvent in the droplet dissolved the polymer at the bottom and, along the side direction at the same time, the silver nanoparticles migrated to the edge of the droplet immediately. With the evaporation of the solvent, forming a hole in the center of the droplet, the residual solvent would not stop the erosion until exhausting the solvent. At last, each droplet would turn into a ring-like structure consisting of the solute with a hole in the center and a gap between adjacent rings. In the model as shown in Figure 7c, two dominant factors prevent the droplets from coalescing: One is the repulsive force which originates from the strong opposite evaporation flux at the edge due to the wedge geometry; Another is the accumulated polymer in the channel, which has been proved in Figure 4b. 

In theory, this strategy would bring diverse possibilities by accommodating the solvent, the solute, the substrate, and the concentration of the solute, and a large variety of different hole geometries can be created. The size of the structure could also be regulated by adjusting the droplet space, using the electrohydrodynamic (EHD) printing technology, which has a smaller nozzle size and a droplet with a size of about several fl, or by increasing the thickness of the polymer layer to increase the solvent consumption in a vertical direction. Estimating possible applications of this structuring technique, it would be templates for functional materials or small containers for biologically and/or technologically relevant materials and may offer a new solution to inkjet-printed OLEDs or quantum dot light-emitting diodes (QLEDs). Hence, with a functional material in the ink, it could obtain orderly, homogeneous microstructures arrays which could not be realized through direct piezoelectric inkjet printing. 

## 4. Conclusions

In summary, we demonstrated the processes of inkjet printing of a mixture of solvent and a solute deposited on a substrate for etching. Due to the coffee-ring effect, the solute was deposited at the edge and left the hole in the center of the droplet. Surprisingly, the adjacent droplets did not coalesce but with a gap, which may be due to the repulsive force which originates from the strong opposite evaporation flux at the edge as a result of the wedge geometry and the accumulated polymer in the channel. This strategy brings us a new way for materials deposition, which could break the limits of traditional direct piezoelectric inkjet printing.

## References

[1] Seifert T., Sowade E., Roscher F., Wiemer M., Baumann R.R. (2015). Additive manufacturing technologies compared: Morphology of deposits of silver ink using inkjet and aerosol jet printing. Ind. Eng. Chem. Res..

[2] Fukuda K., Sekine T., Kumaki D., Tokito S. (2013). Profile control of inkjet printed silver electrodes and their application to organic transistors. ACS Appl. Mater. Inter..

[3] Ning H., Tao R., Fang Z., Cai W., Chen J., Zhou Y., Zhu Z., Zheng Z., Yao R., Xu M. (2017). Direct patterning of silver electrodes with 2.4 μm channel length by piezoelectric inkjet printing. J. Colloid. Interf. Sci..

[4] Kwon J., Lee D., Oh J. (2018). Formation and characterization of inkjet-printed nanosilver lines on plasma-treated glass substrates. Appl. Sci..

[5] Ning H., Chen J., Fang Z., Tao R., Cai W., Yao R., Hu S., Zhu Z., Zhou Y., Yang C. (2017). Direct inkjet printing of silver source/drain electrodes on an amorphous ingazno layer for thin-film transistors. Materials.

[6] Matsuzaki S., Nobusa Y., Yanagi K., Kataura H., Takenobu T. (2011). Inkjet printing of carbon nanotube complementary inverters. Appl. Phys. Express.

[7] Lan M., Hu Z., Zhong Z., Jiang C., Jian W., Peng J., Yong C. (2017). Inkjet-printing line film with varied droplet-spacing. Org. Electron..

[8] Lange A., Wegener M., Boeffel C., Fischer B., Wedel A., Neher D. (2010). A new approach to the solvent system for inkjet-printed P3HT: PCBM solar cells and its use in devices with printed passive and active layers. Sol. Energy Mater. Sol. Cells.

[9] Giuri A., Saleh E., Listorti A., Colella S., Rizzo A., Tuck C., Esposito Corcione C. (2019). Rheological tunability of perovskite precursor solutions: From spin coating to inkjet printing process. Nanomater. Basel.

[10] Oliveira J., Correia V., Sowade E., Etxebarria I., Rodriguez R.D., Mitra K.Y., Baumann R.R., Lancerosméndez S. (2018). Indirect X-ray detectors based on inkjet-printed photodetectors with a screen-printed scintillator layer. ACS Appl. Mater. Interfaces.

[11] Deegan R.D., Bakajin O., Dupont T.F., Huber G., Nagel S.R., Witten T.A. (1997). Capillary flow as the cause of ring stains from dried liquid drops. Nature.

[12] Zhiliang Z., Xingye Z., Zhiqing X., Mengmeng D., Yongqiang W., Yanlin S. (2013). Controlled inkjetting of a conductive pattern of silver nanoparticles based on the coffee-ring effect. Adv. Mater..

[13] Zhang Z., Zhu W. (2014). Controllable fabrication of a flexible transparent metallic grid conductor based on the coffee ring effect. J. Mater. Chem. C.

[14] Chen R., Zhang L., Li X., Ong L., Soe Y.G., Sinsua N., Gras S.L., Tabor R.F., Wang X., Shen W. (2017). Trace analysis and chemical identification on cellulose nanofibers-textured sers substrates using the “coffee ring” effect. ACS Sens..

[15] Layani M., Gruchko M., Milo O., Balberg I., Azulay D., Magdassi S. (2009). Transparent conductive coatings by printing coffee ring arrays obtained at room temperature. ACS Nano.

[16] Li Y., Lan L., Xiao P., Sun S., Lin Z., Song W., Song E., Gao P., Wu W., Peng J. (2016). Coffee-ring defined short channels for inkjet-printed metal oxide thin-film transistors. ACS Appl. Mater. Inter..

[17] Chen F.C., Lu J.P., Huang W.K. (2009). Using ink-jet printing and coffee ring effect to fabricate refractive microlens arrays. IEEE Photonics Technol. Lett..

[18] Li Y., Zhang W., Hu J., Wang Y., Feng X., Du W., Guo M., Liu B. (2017). Rapid assembly of large scale transparent circuit arrays using pdms nanofilm shaped coffee ring. Adv. Funct. Mater..

[19] Hyun Suk K., Cheol Hee L., Sudeep P.K., Todd E., Crosby A.J. (2010). Nanoparticle stripes, grids, and ribbons produced by flow coating. Adv. Mater..

[20] Yabu H., Shimomura M. (2005). Preparation of self-organized mesoscale polymer patterns on a solid substrate: Continuous pattern formation from a receding meniscus. Adv. Funct. Mater..

[21] Chandra D., Yang S. (2009). Capillary-force-induced clustering of micropillar arrays: Is it caused by isolated capillary bridges or by the lateral capillary meniscus interaction force?. Langmuir.

[22] Chen P., Huang Y., Bhave G., Hoshino K., Zhang X. (2016). Inkjet-print micromagnet array on glass slides for immunomagnetic enrichment of circulating tumor cells. Ann. Biomed. Eng..

[23] de Gans B.J., Hoeppener S., Schubert U.S. (2006). Polymer-relief microstructures by inkjet etching. Adv. Mater..

[24] Kawase T., Sirringhaus H., Friend R.H., Shimoda T. (2010). Inkjet printed via-hole interconnections and resistors for all-polymer transistor circuits. Adv. Mater..

[25] Sirringhaus H., Kawase T., Friend R.H., Shimoda T., Inbasekaran M., Wu W., Woo E.P. (2000). High-resolution inkjet printing of all-polymer transistor circuits. Science.

[26] Zhang L., Liu H., Zhao Y., Sun X., Wen Y., Guo Y., Gao X., Di C., Yu G., Liu Y. (2012). Inkjet printing high-resolution, large-area graphene patterns by coffee-ring lithography. Adv. Mater..

[27] Stringer J., Derby B. (2009). Limits to feature size and resolution in ink jet printing. J. Eur. Ceram. Soc..

[28] Fukuda K., Someya T. (2017). Recent progress in the development of printed thin-film transistors and circuits with high-resolution printing technology. Adv. Mater..

[29] Wang H., Cheng C., Zhang L., Liu H., Zhao Y., Guo Y., Hu W., Yu G., Liu Y. (2014). Inkjet printing short-channel polymer transistors with high-performance and ultrahigh photoresponsivity. Adv. Mater..

